# [Retracted] The role of quercetin and vitamin C in Nrf2-dependent oxidative stress production in breast cancer cells

**DOI:** 10.3892/ol.2025.15124

**Published:** 2025-06-02

**Authors:** Zohreh Mostafavi‑Pour, Fatemeh Ramezani, Fatemeh Keshavarzi, Nasser Samadi

Oncol Lett 13: 1965–1973, 2017; DOI: 10.3892/ol.2017.5619

Following the publication of the above paper, a concerned reader drew the Editor's attention to the fact that, regarding the western blot data shown in Fig. 3D on p. 1970, there appeared to be a number of strikingly similar protein bands featured in all the rows of data in this figure, in addition to certain bands that bore striking resemblance to each other, but which were proportioned differently, such that the similarities were difficult to attribute to coincidence.

Following an internal enquiry, the Editor of *Oncology Letters* was able to verify the claims made by the concerned reader; therefore, the Editor has decided that the paper should be retracted from the Journal on account of a lack of confidence in the presented data. The authors were asked for an explanation to account for these concerns, but the Editorial Office did not receive a reply. The Editor apologizes to the readership of the Journal for any inconvenience caused.

